# Efficient NADPH-dependent dehalogenation afforded by a self-sufficient reductive dehalogenase

**DOI:** 10.1016/j.jbc.2023.105086

**Published:** 2023-07-25

**Authors:** Karl Fisher, Tom Halliwell, Karl A.P. Payne, Gabriel Ragala, Sam Hay, Stephen E.J. Rigby, David Leys

**Affiliations:** Manchester Institute of Biotechnology, University of Manchester, Manchester, UK

**Keywords:** reductive dehalogenase, bioremediation, cobalamin, B12, Fe-S clusters, EPR

## Abstract

Reductive dehalogenases are corrinoid and iron–sulfur cluster–containing enzymes that catalyze the reductive removal of a halogen atom. The oxygen-sensitive and membrane-associated nature of the respiratory reductive dehalogenases has hindered their detailed kinetic study. In contrast, the evolutionarily related catabolic reductive dehalogenases are oxygen tolerant, with those that are naturally fused to a reductase domain with similarity to phthalate dioxygenase presenting attractive targets for further study. We present efficient heterologous expression of a self-sufficient catabolic reductive dehalogenase from *Jhaorihella thermophila* in *Escherichia coli*. Combining the use of maltose-binding protein as a solubility-enhancing tag with the *btuCEDFB* cobalamin uptake system affords up to 40% cobalamin occupancy and a full complement of iron–sulfur clusters. The enzyme is able to efficiently perform NADPH-dependent dehalogenation of brominated and iodinated phenolic compounds, including the flame retardant tetrabromobisphenol, under both anaerobic and aerobic conditions. NADPH consumption is tightly coupled to product formation. Surprisingly, corresponding chlorinated compounds only act as competitive inhibitors. Electron paramagnetic resonance spectroscopy reveals loss of the Co(II) signal observed in the resting state of the enzyme under steady-state conditions, suggesting accumulation of Co(I)/(III) species prior to the rate-limiting step. *In vivo* reductive debromination activity is readily observed, and when the enzyme is expressed in *E. coli* strain W, supports growth on 3-bromo-4-hydroxyphenylacetic as a sole carbon source. This demonstrates the potential for catabolic reductive dehalogenases for future application in bioremediation.

The production of halogenated organic compounds (organohalides) is fundamental to a variety of industrial applications, including their use as solvents, degreasing agents, pesticides, and flame retardants and as intermediates for chemical synthesis. As a consequence, their presence in the environment has increased significantly over the past 100 years. Organohalides are now one of the largest groups of environmentally prevalent recalcitrant and carcinogenic compounds that pollute both our soil and groundwater (1). However, organohalide respiring bacteria can utilize such compounds as terminal electron acceptors under anaerobic conditions, through a process known as organohalide respiration. This process provides a realistic solution to remediate contaminated sites. Organohalide respiration in these bacteria is driven by the reductive dehalogenase enzymes acting as terminal electron acceptor (RdhAs) (2).

The RdhAs are cobalamin and iron–sulfur cluster–containing enzymes that catalyze the reductive dehalogenation of organohalide compounds. Along with the epoxyqueuosine reductases, they form the class III family of the cobalamin-dependent enzymes (3, 4, 5). The RdhAs initially proved resistant to recombinant expression in standard expression systems (6), and biochemical characterization relied on what little native material could be purified from dehalorespiring bacteria. Recently, overexpression of recombinant reductive dehalogenases was achieved either through expression systems within dehalorespiring bacteria or through heterologous expression in nonstandard expression systems (4, 5). These recent developments led to the elucidation of crystal structures of two RdhAs, the respiratory PceA from *Dehalospirillum multivorans* (7) and a catabolic RdhA from *Nitratireductor pacificus* (8, 9).

The reductive dehalogenases can be divided into four subfamilies based on their domain organization (Fig. 1). Two subfamilies, which differ due to the presence/absence of a reductase domain, form what have become known as the catabolic RdhAs. The other two subfamilies form the respiratory and the transmembrane reductive dehalogenases.

**Figure 1:**
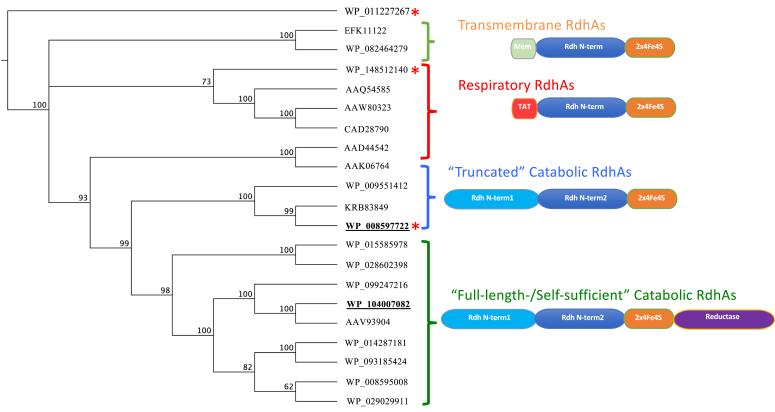
**Phylogenetic analysis of reductive dehalogenases.** Phylogenetic tree with branches grouped according to their respective subfamilies and the domain organization of each subfamily indicated. Only the sequence corresponding to the functional cobalamin (*dark blue*) and iron-sulfur–binding domains (*orange*) are included for the RdhA alignment. *Red asterisks* indicate those homologues for which crystal structures exist and homologues used in this study are underlined in *bold*. Genes used include EFK11122, delta proteobacterium NaphS2 RdhA; WP_082464279, *Dethiosulfatarculus sandiegensis* RdhA; WP_148512140, *Dehalospirillum multivorans* PceA; AAQ54585, *Desulfitobacterium hafniense* PCP-1 CprA5; AAW80323, *Desulfitobacterium hafniense* Y51 PceA; CAD28790, *Dehalobacter restrictus* PceA; AAD44542, *Desulfitobacterium dehalogenans* ATCC 51507 CprA; AAK06764, *Desulfitobacterium hafniense* PCP-1 CprA3; WP_009551412, *Burkholderiales* bacterium JOSHI_001 RdhA; KRB83849, *Noviherbaspirillum* sp. Root189 RdhA; WP_008597722, *Nitratireductor pacificus* RdhA3; WP_015585978,= *Comamonas sp*. 7D-2 BhbA; WP_028602398, *Ottowia thiooxydans* RdhA; WP_099247216, *Tropicibacter phthalicicus* RdhA; WP_104007082, *Jhaorihella thermophila* RdhA; AAV93904, *Ruegeria pomeroyi* DSS-3 RdhA; WP_014287181, *Pseudovibrio* sp. FO-BEG1 RdhA; WP_093185424, *Pseudovibrio* sp. Tun.PSC04-5.I4 RdhA; WP_008595008, *Nitratireductor pacificus* RdhA2; WP_029029911, *Salinarimonas rosea* RdhA. The tree was rooted with *Streptococcus thermophilus* QueG (WP_011227267).

The first catabolic RdhA to be described, BhbA, is an enzyme involved in the degradation of the herbicide bromoxynil in *Comamonas* sp. 7D-2 (10). The gene encoding BhbA is found within a gene cluster on a plasmid encoding all of the proteins that are required for the aerobic degradation of bromoxynil to 4-carboxy-2-hydroxymuconate-6-semialdehyde, which then feeds into the tricarboxylic acid cycle cycle. As such bromoxynil fulfills a role as a carbon source rather than as a terminal electron acceptor.

The catabolic RdhAs are distinct from the respiratory RdhAs by the fact they possess a vestigial duplication of the cobalamin-binding core domain. The crystal structure of the *N. pacificus* RdhA (*Np*RdhA) shows that this additional vestigial domain, which lacks the ability to bind cobalamin, forms an interface with the cobalamin-containing core domain mimicking the monomer–monomer interface observed for the respiratory RdhA dimer. As a result, *Np*RdhA is monomeric, in contrast to the dimeric *D. multivorans* PceA (7, 8). The catabolic RdhAs group with the *ortho*-chlorophenol branch of the respiratory dehalogenases (Fig. 1). All catabolic RdhAs characterized to date have shown specificity for ortho-halogenated phenol substrates (8, 10).

Most catabolic RdhAs, such as BhbA, possess a C-terminal phthalate dioxygenase reductase (PDOR) domain that presumably allows electrons derived from NAD(P)H to drive reductive dehalogenation. These RdhAs are therefore labeled “full-length/self-sufficient” as they do not require redox partner proteins for dehalogenation. There is, however, a clade of catabolic RdhAs that lack this reductase domain. In some cases, such as *Np*RdhA, these naturally “truncated” catabolic RdhAs are located in a gene cluster with genes encoding a ferredoxin oxidoreductase and two ferredoxins. While this genomic context hints at a plausible route for supplying electrons to the RdhA, attempts to reconstitute this pathway *in vitro* with recombinant proteins failed to show dehalogenase activity (11). However, dehalogenation could be achieved *in vitro* using a nonphysiological system composed of spinach ferredoxin and an *Escherichia coli* flavodoxin reductase. When all three genes were heterologously expressed in the soil bacterium, *Bacillus megaterium, Np*RdhA dehalogenase activity could also be detected *in vivo*. However, the three components in this system lack specificity for one another making both detailed study as well as application impractical. The full-length/self-sufficient catabolic RdhAs present a preferable model system both for future bioremediation purposes as well as detailed mechanistic studies. However, the expression of full-length self-sufficient catabolic reductive dehalogenases has previously resulted in either insoluble protein or small quantities of soluble protein mostly devoid of cofactors and activity (11, 12). We selected a self-sufficient catabolic reductive dehalogenase RdhA from *Jhaorihella thermophila* (*Jt*RdhA), a moderately thermophilic marine bacterium (13) and report successful heterologous expression of the full-length enzyme in *E. coli* HMS-174 utilizing the *btu* vitamin B12 salvage pathway that consists of a series of transporter proteins (BtuCEDFB) for cobalamin uptake (14).

## Results

### Computational modeling of *J*. *thermophila RdhA* structure

**Figure 2:**
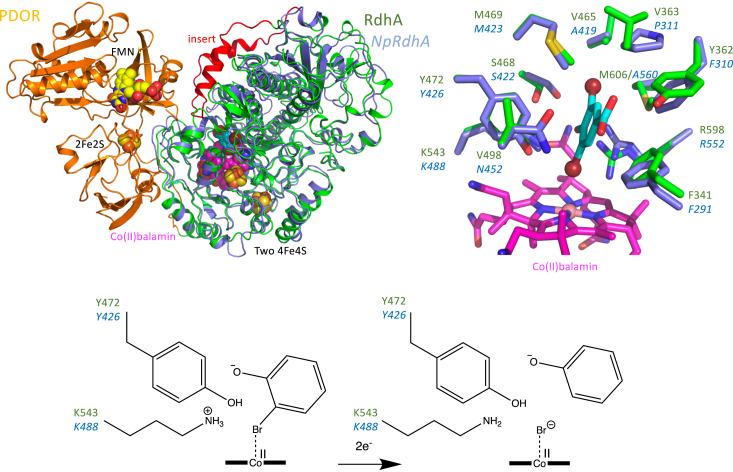
**AlphaFold model of *Jt*RdhA.***Top left*, an overlay of the *Jt*RdhA alphamodel (RdhA domain in green, PDOR domain in *orange*) with cofactors added (shown in atom *colored spheres*) with the related *Np*RdhA (in *blue*). An insert specific to *Jt*RdhA and related RdhA-PDOR fusions is shown in *red*. *Top right* depicts a detailed overlay of the *Np*RdhA active site (key residues shown in atom *colored sticks* with *blue* carbons) with the corresponding 3,5-dibromo-4-hydroxybenzoic acid substrate (*cyan* carbons) and the *Jt*RdhA model (*green* carbons). The proposed overall reaction for a bromophenolic substrate is depicted at the *bottom*, with proton transfer from the conserved Y-K/R dyad coupled to electron transfer to the substrate.

*J. thermophila* is a moderately thermophilic, gram-negative bacterium that was isolated from a coastal hot spring near Taiwan (13). From genome analysis a putative self-sufficient reductive dehalogenase was identified with similar features to *Np*RdhA (absence of TAT signal, a duplicated cobalamin-binding domain, and a 2 [4Fe-4S] binding sequence) fused to a PDOR-like domain. Sequence alignment of *Jt*RdhA with *Np*RdhA shows ∼39% sequence identity with the key residues involved in *Np*RdhA substrate binding and catalysis (*i.e.*, Y426, K488, R552) present in *Jt*RdhA. AlphaFold modeling combined with placement of the various cofactors (corrinoid, two 4Fe4S, 2FeS, and FMN) leads to a reasonable model for the full-length self-sufficient enzyme. This suggests an insertion in the RdhA domain (compared with NpRdhA) folds over the active site (Fig. 2) and might be involved in domain interactions with the PDOR module. While the exact interaction of the individual domains is likely the least accurate component of the model, domain motion of the 2Fe2S could allow electron transfer from NAD(P)H *via* the FMN to the 2x[4Fe-4S]/corrinoid cofactors.

### Heterologous expression of *J. thermophila RdhA*

**Figure 3:**
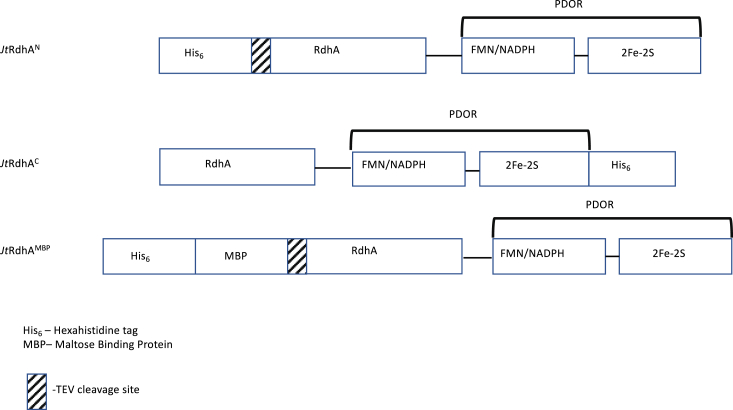
***Jhaorihella thermophila* RdhA constructs.** Constructs were designed for the expression of *Jt*RdhA including a hexahistidine tag and maltose-binding protein.

Synthesis of the *Jt*RdhA gene was performed for expression in *E. coli* HMS-174 (DE3) after previous studies showed good levels of protein expression and cobalamin incorporation in that strain (up to ∼2.6 mg purified protein per gram cell weight and 30% B12 occupancy) (5). The *Jt*RdhA gene was cloned into suitable vectors to allow the screening of constructs containing purification and solubility tags to produce soluble and active protein. These tags included both N- and C-terminal hexahistidine tags (*Jt*RdhA^N^ and *Jt*RdhA^C^, respectively) and an N-terminal His-maltose binding protein-TEV tag (*Jt*RdhA^MBP^), to induce increased solubility (Fig. 3). Cells were cotransformed with the recently reported pBAD-BtuCEDFB plasmid (15) to provide high cobalamin availability during protein expression.

### Purification and enzyme activity analysis of *Jt*RdhA^C^ and *Jt*RdhA^N^

Anaerobic purification of *Jt*RdhA^C^ from *E. coli* HMS174(DE3) cells coexpressed with the cobalamin transporter BtuCEDFB resulted in the majority of the protein being present in the insoluble fraction. Anaerobic purification of JtRdhA^N^, however, was successful with a band ∼120 kDa present in both the 40 mM wash and elution fractions (Fig. S1). Fractions containing the protein of interest were pooled and analyzed by SDS-PAGE with the purity of the final enriched enzyme judged to be greater than 85% and with a yield of ∼43 mg eluted protein from 100 g cell weight. *Jt*RdhA^N^ activity measured from NADPH consumption at 340 nm in an anaerobic glove box at 25 °C was 240 ± 8 μM/min/μM *Jt*RdhA with 35-DB-4-OH as substrate. Product formation from 35-DB-4-OH was unchanged when the spinach ferredoxin noncognate reductase system was included in steady-state assays, suggesting a full complement of Fe-S clusters in the *Jt*RdhA^N^ enzyme. The study of N-terminally tagged *Jt*RdhA^N^ was therefore continued in preference over the C-terminal tagged protein.

### Increased solubility of *Jt*RdhA^N^ by fusion to maltose-binding protein

Owing to the relatively low recovery levels of purified *Jt*RdhA^N^, an N-terminal fusion protein with maltose-binding protein (MBP) was tested for improved protein yields. Anaerobic purification of *Jt*RdhA^MBP^ was performed from *E. coli* HMS-174 cells using a Ni-NTA nickel column (Fig. S2*A*). As observed for the purification of previously described constructs, binding to the Ni-NTA column was weak with a protein band corresponding to *Jt*RdhA^MBP^ in both the 15 and 30 mM imidazole wash fractions. However, the elution fractions contained a substantially increased quantity of *Jt*RdhA^MBP^ compared with the previous *Jt*RdhA^N^ yields, 127 mg protein from 100 g cell weight compared with 43 mg, respectively, confirming that fusion to MBP significantly increased protein yields. Further purification *via* amylose resin chromatography allowing affinity purification of MBP surprisingly did not improve the purity of *Jt*RdhA^MBP^ (data not shown). However, size-exclusion chromatography of the Ni-NTA nickel column elution fractions allowed further purification (Fig. S2*B*). *Jt*RdhA^MBP^ activity measured from NADPH consumption at 340 nm at 25 °C was 258 ± 14 μM/min/μM *Jt*RdhA^MBP^ protein with 35-DB-4-OH as substrate. The NADPH-dependent 35-DB-4-OH reductase activity of *Jt*RdhA^MBP^ in the presence and absence of the noncognate spinach ferredoxin reductase system was unchanged, again suggesting a full complement of Fe-S clusters in the *Jt*RdhA^MBP^. Given the increased yields of *Jt*RdhA^MBP^ in comparison with *Jt* RdhA^N^, further studies were carried out with *Jt*RdhA^MBP^ unless otherwise stated.

### Characterization of *Jt*RdhA^MBP^ cofactors

**Figure 4:**
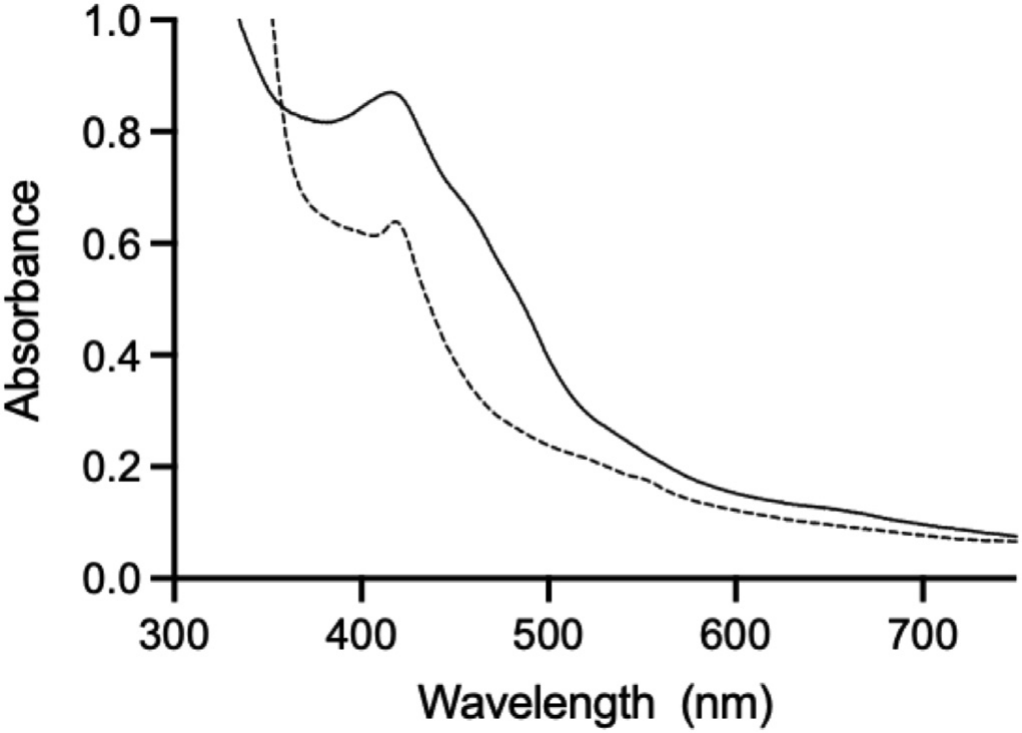
**UV-visible spectra of *Jt*RdhA**^**MBP**^**.***Jt*RdhA^MBP^ (25 μM) as isolated (*solid line*) and reduced achieved by addition of 1 mM sodium dithionite (*broken line*).

Purified protein used for characterization contained 0.4 ± 0.07 cobalamin, 0.53 ± 0.042 FMN, and 9.4 ± 0.1 irons (2x [4Fe-4S] and 1x [2Fe-2S] clusters) per *Jt*RdhA^MBP^. Protein concentration was determined by the Lowry method (16). The UV-visible spectrum of *Jt*RdhA^MBP^ exhibits a broad absorbance with features in the 450- to 410-nm region presumably due to the presence of FMN and iron–sulfur clusters in the protein, respectively (Fig. 4), and is comparable with that previously reported for the *Np*RdhA protein (8). Upon dithionite reduction, these features are partially bleached because FMN and Fe-S clusters are reduced.

### Comparison of kinetic parameters for *Jt*RdhA^MBP^-catalyzed substrate reduction

**Figure 5:**
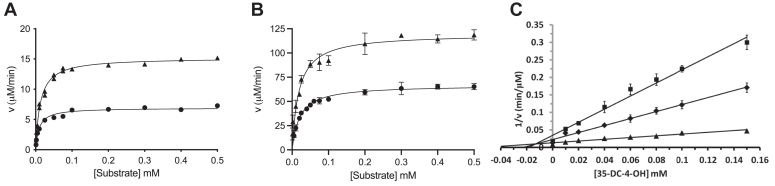
**Kinetic analysis of NADPH-driven *Jt*RdhA**^**MBP**^**activity with 3,5-dibromo-4-hydroxybenzoic and 3-bromo-4-hydroxybenzoic acid.***Jt*RdhA^MBP^ NADPH oxidation activity as a function of substrate concentration employing enzyme from cells cotransformed with BtuB (*A*) and BtuCEDFB (*B*). Assays contain 200 μM NADPH and 0.01 to 0.5 mM substrate. Assays were started by the addition of 0.5 μM or 1 μM *Jt*RdhA^MBP^ for 3,5-dibromo-4-hydroxybenzoic acid (▲) and 3-bromo-4-hydroxybenzoic acid (●), respectively. *C*, dixon plot showing the inhibition of *Jt*RdhA^MBP^ activity with 3,5-dibromo-4-hydroxybenzoic acid at three concentrations, 0.015 mM (■), 0.03 mM (♦), and 0.1 mM (▲), by 3,5-dichloro-4-hydroxybenzoic acid.

Heterologous expression of *Jt*RdhA^MBP^ in *E. coli* HMS-174 (DE3) allowed the production of sufficient quantities of protein for characterization by various techniques with the same batch of purified protein. It was previously shown that *Np*RdhA prefers 35-DB-4-OH over 3-bromo-4-hydroxybenzoic acid (3-B-4-OH) (8, 11); hence, the ability of *Jt*RdhA^MBP^ to perform dehalogenation of both substrates was measured and the importance of BtuCEDFB coexpression investigated (Fig. 5). *Jt*RdhA^MBP^ purified from cells coexpressed with BtuB (*Jt*RdhA^MBP^-BtuB) exhibited a mild preference for 35-DB-4-OH with an apparent k_cat_ of 15.2 ± 0.3 min^−1^ in comparison with 6.9 ± 0.2 min^−1^ for 3-B-4-OH. *Jt*RdhA^MBP^-BtuB has a K_M_ of 13 ± 0.5 μM and 9 ± 0.4 μM for 35-DB-4-OH and 3-B-4-OH, respectively (Fig. 5*A*). It was not possible to accurately determine cobalamin content due to low levels of incorporation. In agreement, *Jt*RdhA^MBP^-BtuCEDFB also showed preference for 35-DB-4-OH with k_cat_ = 120 ± 3 min^−1^ in comparison with k_cat_ = 66.7 ± 0.6 min^−1^ for 3-B-4-OH (Fig. 5*B*; 600 ± 8 min^−1^ and 164 ± 2 min^−1^, respectively, when calculated using cobalamin concentration). *Jt*RdhA^MBP^-BtuCEDFB has a K_M_ of 19 ± 0.7 μM and 21 ± 0.2 μM for 35-DB-4-OH and 3-B-4-OH, respectively.

### *Jt*RdhA^MBP^ substrate scope

Initial analysis of the purified *Jt*RdhA^MBP^ confirms it can efficiently perform NADPH-dependent dehalogenation on a variety of brominated compounds. The relative rate of substrate utilization, as an indication of substrate scope, by *Jt*RdhA^MBP^ with a range of ortho-phenol brominated and chlorinated compounds in a standard assay is shown in Table 1. Substrate turnover was measured by NADPH consumption and confirmed with HPLC analysis. *Jt*RdhA^MBP^ has clearly defined substrate preferences and can dehalogenate a variety of brominated compounds but not chlorinated or fluorinated compounds; similar observations were made for the recently reported tetrabromobisphenol A (TBBPA) dehalogenase from *Ochrobactrum* sp. T (17). This substrate range was confirmed with assays utilizing reduced methyl viologen as electron donor or the noncognate spinach ferredoxin and *E. coli* flavodoxin reductase electron transfer system to circumvent the internal RdhA electron transfer mechanism. While 3-bromo-4-hydroxy phenyl acetic acid exhibited activity comparable with that of 35-DB-4-OH, which was the best of the brominated substrates tested, 3-bromo-4-hydroxy benzoic acid exhibited approximately 50% maximum measured activity. In addition, iodinated compounds exhibited good turnover activity with 3,5 diiodo-4-hydroxybenzoic acid, 2-iodophenol, and 2,4,6-triiodophenol, operating at approximately 84%, 72%, and 66% the rate of that observed with 35DB-4-OH, respectively (Table 1). The chlorinated organohalides investigated (see legend to Table 1) all inhibited 35-DB-4-OH reduction activity, suggesting they could bind nonproductively to the active site. The effect of 35-DC-4-OH (the chloro-analogue of 35-DB-4-OH) on NADPH oxidation activity with 35-DB-4-OH as substrate was investigated quantitatively, yielding a K_i_ of 13.7 ± 3.6 μM and competitive inhibition (Fig. 4*C*). While these experiments are difficult at nonsaturating substrate concentrations, the V_max_ determined from the Dixon plot (112 ± 12 μM/min) (18) is consistent with the kinetic parameters detailed in the previous section.

**Table 1:** Relative rate of substrate dehalogenation by *Jt*RdhA^MBP^

Substrate	Structure	NADPH consumption μM min^−1^	% Maximum rate
3,5-Dibromo-4-hydroxy benzoic acid		148 ± 1	100
3-Bromo-4-hydroxy phenyl acetic acid		147 ± 3	99.3
3-Bromo-4-hydroxy benzoic acid		73 ± 6	49.7
2-Bromo-4-nitrophenol		28 ± 2	19.2
Bromoxynil		20 ± 1	13.6
2,6-Dibromo-4-methyl phenol		12 ± 1	8.2
2,6-Dibromophenol		10 ± 1	6.8
3,5 Diiodo-4-hydroxybenzoic acid		124 ± 8	84
2-Iodophenol		106 ± 3	71.6
2,4,6-Triiodophenol		97 ± 14	65.5

*Jt*RdhA^MBP^ activity was measured with various brominated, iodinated, and chlorinated substrates by monitoring NADPH consumption at 340 nm under an N_2_ atmosphere at 20 ° C. All reactions were performed in triplicate and contained 0.5 μM *Jt*RdhA^MBP^ protein and 0.2 mM NADPH and 0.25 mM substrate. Results were confirmed by HPLC analysis with standards allowing identification of product peaks where possible. No dehalogenation activity was measured with 5-bromo-2-furoic acid, 3-chloro-4-hydroxyphenyl acetic acid, 3-chloro-4-hydroxy benzoic acid, 3,5-dichloro-4-hydroxy benzoic acid, 2-chloro-4-nitrophenol, 3-chloro-phenylacetic acid, 4-chloro-benzoic acid, 3,4-dichloro-phenol, 3-fluoro-4-hydroxyphenylacetic acid, 3-fluoro-4-hydroxybenzoic acid, 3-fluoro-4-nitrophenol, 2,6-difluorophenol, and 3-fluorophenyl acetic acid. The variety of substrates tested was limited to commercially available compounds.

**Figure 6:**
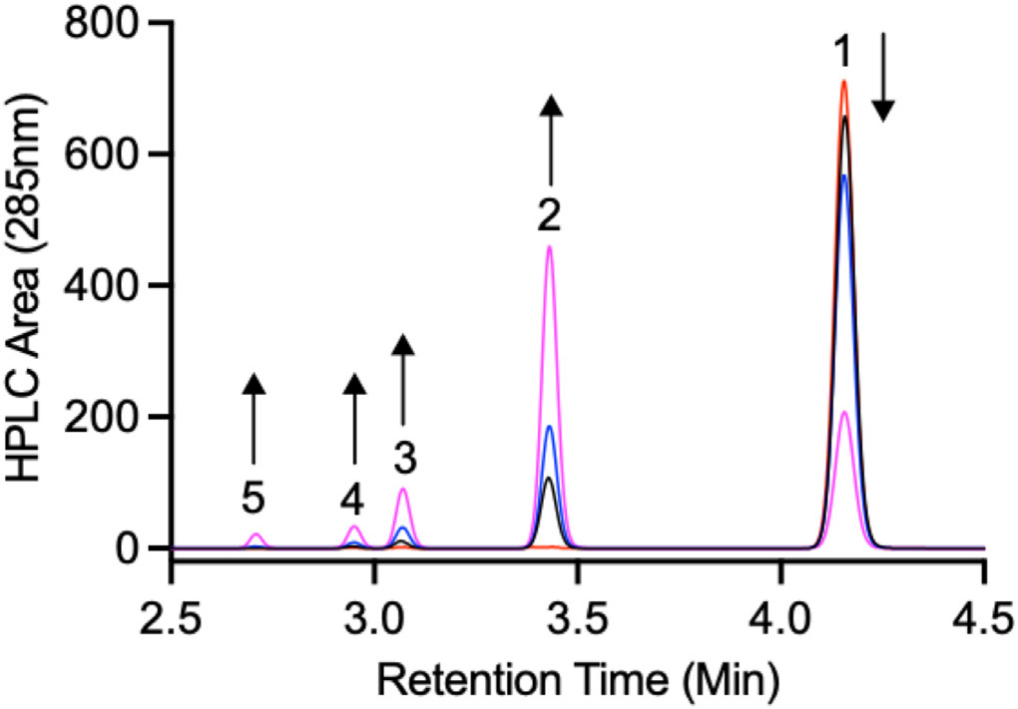
**Time-dependent dehalogenation of tetrabromobisphenol A with *Jt*RdhA**^**MBP**^**.** TBBP dehalogenation was performed under an N_2_ environment with reactions *left* shaking at 30 °C. TBBP (2 mM) was dissolved in anaerobic iso-octane, and assays were performed as biphasic reactions. Reaction samples were taken after 0 (*red*), 2 (*black*), 5 (*blue*), and 20 h (*magenta*), and LC-MS analysis was performed on the organic phase measuring absorbance at 285 nm. No product was observed in the aqueous phase. The decrease in TBBP (1) concentration was accompanied by the appearance of tribrominated (2), dibrominated (3), and monobrominated (4) intermediates and a small amount of bisphenol A (5). TBBP, tetrabromobisphenol.

It is noteworthy that one of the most commonly used flame retardants TBBPA was determined to be a substrate for *Jt*RdhA^MBP^(Fig. 6). Using biphasic assays, because of the low solubility of TBBPA in water, we could monitor the time-dependent decrease in TBBPA and after 20 h approximately 70% of the starting material (2 mM) had been converted to tri-, di-, and mono-brominated intermediates and bisphenol A.

### Coupling of NADPH oxidation to 3-B-4-OH formation

**Figure 7:**
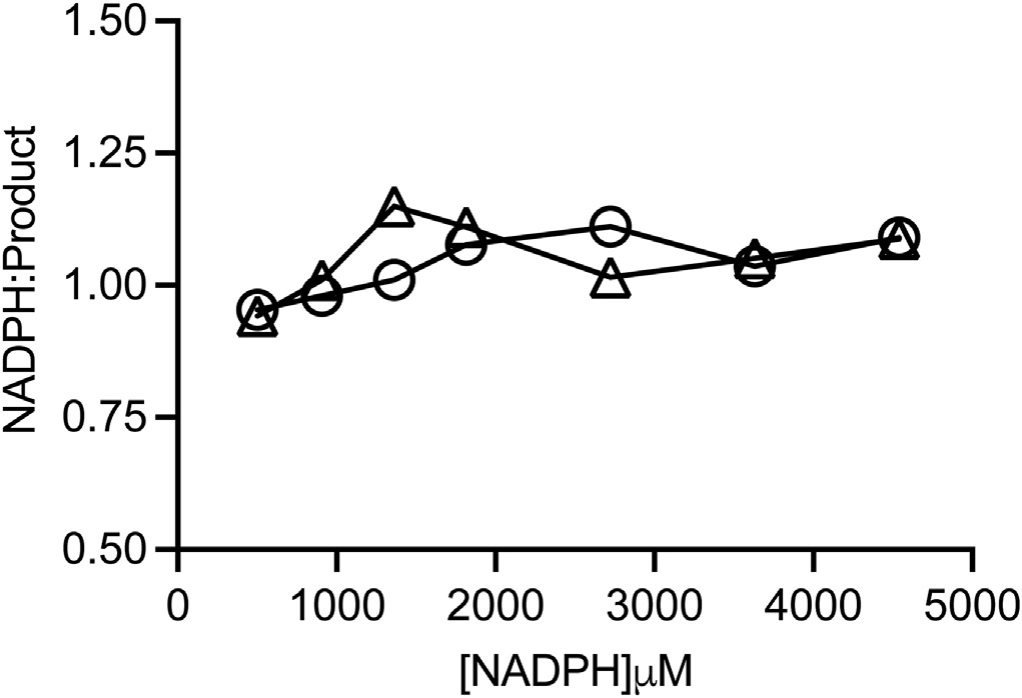
**Coupling of NADPH hydrolysis to *Jt*RdhA**^**MBP**^**-catalyzed product formation.** Measurement of *Jt*RdhA^MBP^ 35DB-4-OH reductase activity was performed in a sealed vial in the presence of either air (Ο) or 100% N_2_ (Δ), under conditions of limiting NADPH. Reactions contained 0.5 μM Jt RdhA protein,10 mM substrate and were *left* shaking overnight at 30 °C to go to completion. Product formation was measured by HPLC analysis, and NADPH concentration was determined prior to reaction start *via* absorbance at 340 nm.

To ascertain the coupling between NADPH oxidation and product formation, under both anaerobic and aerobic conditions, we compared the levels of product formation with respect to NADPH consumption from end point assays under aerobic and anaerobic conditions (Fig. 7). Product formation increased linearly with NADPH concentration, and there was no inhibition of enzyme activity in the presence of oxygen with NADPH usage tightly coupled to product formation. The NADPH:product ratio was calculated as 1.0 ± 0.06 under both aerobic and anaerobic conditions indicating no futile electron transfer. *Jt*RdhA^MBP^ activity remained stable for more than 240 min at 30 °C, indicating oxygen does not inactivate the enzyme.

Although we have shown 35-DC-4-OH is not a substrate and that 35-DB-4-OH binding stops futile electron transfer to oxygen, we did observe that *Jt*RdhA^MBP^ was able to donate electrons to oxygen in the presence of 35-DC-4-OH, albeit at a very slow rate (1.12 μM/min under air compared with 0.15 μM/min under nitrogen; Fig. S3). To investigate this further, we repeated these assays and increased the *Jt*RdhA^MBP^ concentration by a factor of 10 to exacerbate any electron transfer event occurring in the presence of 35-DC-4-OH. In the absence of *Jt*RdhA^MBP^ the rate of NADPH oxidation was 0.02 μM/min, and this increased significantly to 30.7 ± 1.4 μM/min when *Jt*RdhA^MBP^ was added (Fig. S4*A*). Interestingly, assays containing 35-DC-4-OH showed a lower rate of 10.4 ± 0.3 μM/min, suggesting ligand binding in the active site affects electron transfer to oxygen. Fig. S4*B* shows that, under anaerobic conditions the rate of NADPH consumption was very slow and essentially the same with and without 35-DC-4-OH (0.1 μM/min and 0.19 μM/min, respectively).

### The effect of NaCl concentration on *Jt*RdhA^MBP^-catalyzed 35-DB-4-OH reduction

**Figure 8:**
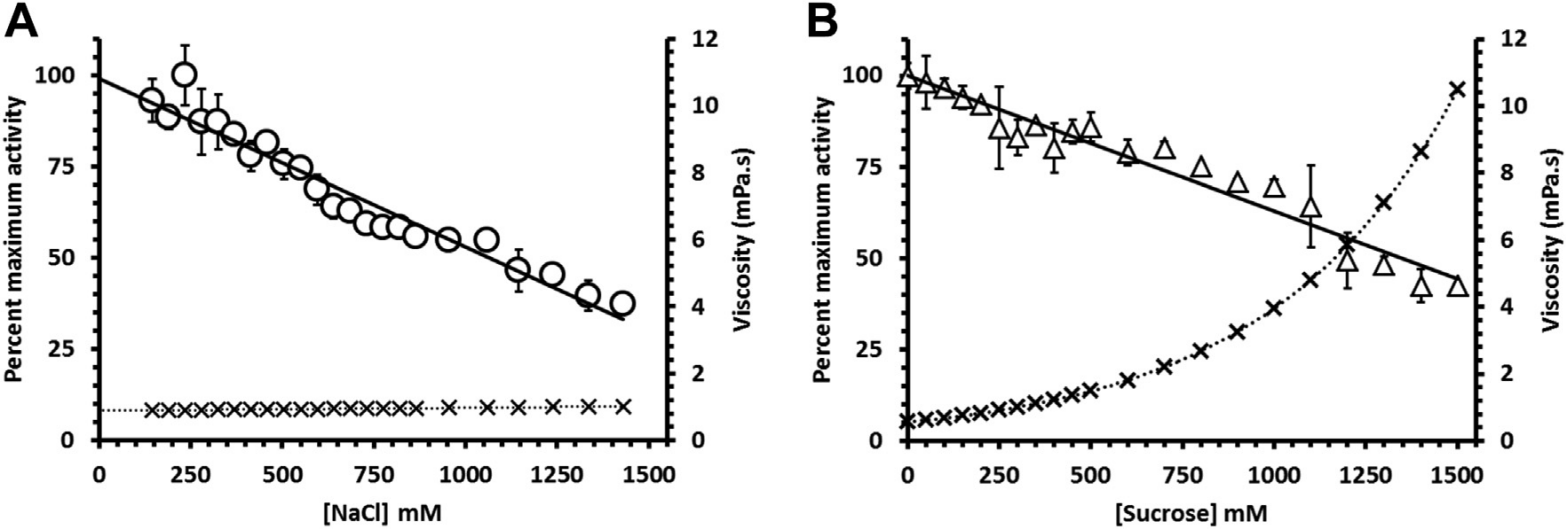
**Viscocity dependentinhibition ofNADPH-driven*****Jt*****RdhA**^**MBP**^**activity.** Assays consisted of 200 μM NADPH, 2 mM 3,5-dibromo-4-hydroxybenzoic acid, 1 μM *Jt*RdhA, and different concentrations of NaCl (*circles*, panel *A*) or sucrose (*triangles*, panel *B*). Each assay, t = 5 min, was performed on a Cary spectrophotometer inside an anaerobic chamber with the absorbance of NADPH measured at 340 nm (ε_340_ = 6.22 mM^−1^ cm^−1^). NaCl concentration was varied from 150 to 1500 mM, and sucrose was varied over the range 50 to 1500 mM. Activity data are an average of triplicates with calculated standard deviation and are fitted to a linear model with Prism 9 software. The viscosities of each solution ( × ) derived from published tables and equations are plotted in mPa.s (right-hand vertical scale) for comparison with the activities including the expected exponential fits.

Salt concentrations above 200 mM have previously been shown to affect the protein–protein interaction of the *Np*RdhA and spinach ferredoxin hybrid system and inhibit the NADPH-dependent dehalogenase activity (11). To determine the optimum ionic strength conditions for *Jt*RdhA^MBP^, we conducted organohalide reductive assays in the presence of various NaCl concentrations. As shown in Figure 8*A*, a linear dependence of enzyme activity on NaCl concentration appeared with 50% inhibition of debromination activity occurring at a NaCl concentration of approximately 1 M. The possibility of viscosity effects affecting enzyme activity, especially at high NaCl concentrations, was controlled for by measuring the activity at increasing viscosities provided by sucrose solutions, Figure 8*B*. The confirmed viscosity can be a factor in reducing the activity of *Jt*RdhA^MBP^, but the viscosities required to do so far exceed those of the NaCl solutions employed (19, 20). This decreased effect of salt concentration on *Jt*RdhA^MBP^ activity relative to *Np*RdhA activity is not altogether surprising as no protein interactions are required for electron transfer to occur, but it is interesting that chloride ion does not compete significantly with the brominated substrate for binding or any interdomain interactions between the RdhA and PDOR modules.

### Electron paramagnetic resonance spectroscopy of *Jt*RdhA^MBP^

**Figure 9:**
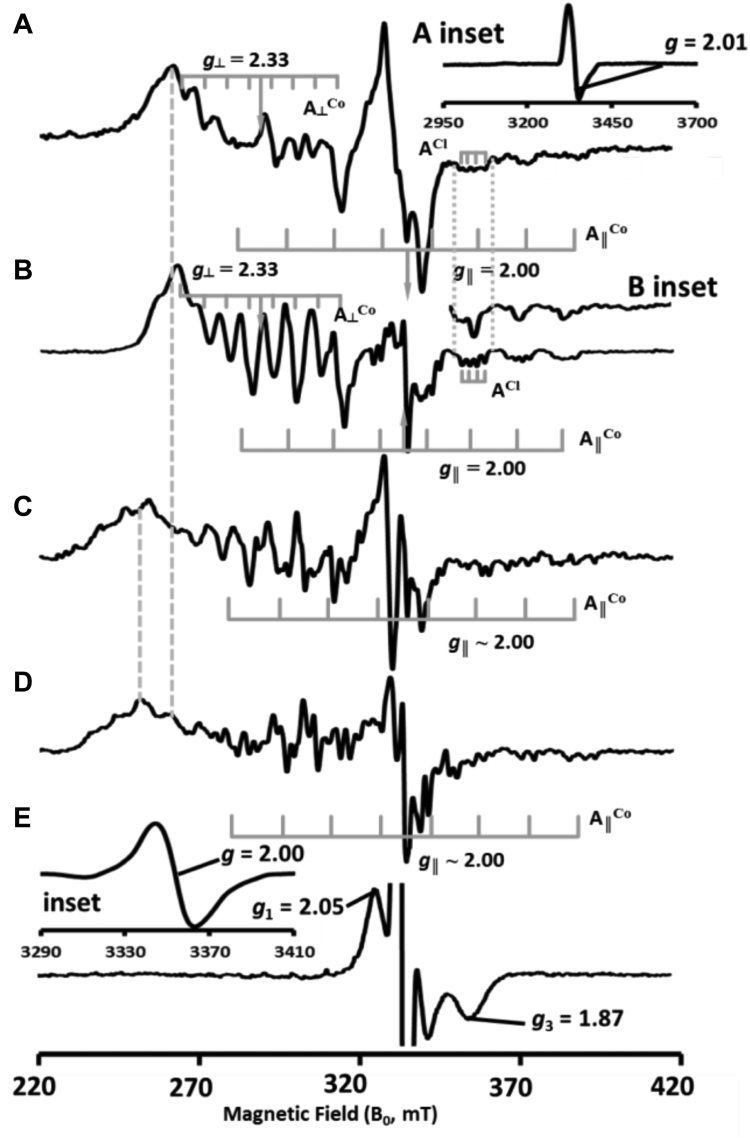
**X-band electron paramagnetic resonance spectra of *Jt*RdhA**^**MBP**^**and comparison with *Np*RdhA.***A*, *Jt*RdhA^MBP^ as isolated in chloride-containing buffer (inset shows contaminating [3Fe-4S]^1+^ signal); *B*, *Np*RdhA as isolated in chloride-containing buffer (inset shows *g*_‖_ region in the absence of chloride); *C*, *Jt*RdhA^MBP^ of A, plus 100-fold excess of 3,5-dibromo-4-hydroxybenzoic acid; *D*, *Np*RdhA of B, plus 100-fold excess of 3,5-dibromo-4-hydroxybenzoic acid; *E*, *Jt*RdhA^MBP^ of C, plus 100-fold excess of NADPH frozen to 77 K while turning over (inset shows narrow sweep, low power spectrum of *g* = 2 region to define radical signal). Experimental parameters: 0.5 mW microwave power, 5 G field modulation, temperature 30 K except inset to E wherein 20 μW microwave power, 1 G field modulation, and 100 K were employed.

The X-band (9–9.5 GHz), continuous-wave electron paramagnetic resonance (EPR) spectrum of *Jt*RdhA^MBP^ at 30 K is shown in Figure 9*A*. It reveals a low-spin, five-coordinate, base-off cob(II)alamin species (21, 22) displaying very similar parameters to those observed for *Np*RdhA (8), Figure 9*B*. However, the *Jt*RdhA^MBP^ exhibits only ∼40% cobalamin incorporation (compared with ∼80% in *Np*RdhA) and is less soluble than *Np*RdhA (due to higher *M*_r_), thus degrading the EPR signal and limiting us to comparing the *Jt*RdhA data with that we published previously for *Np*RdhA. The single unpaired electron in the cob(II)alamin *d*_z_2 orbital (electronic configuration [Ar]3d^7^) combined with I = 7/2 nuclear spin gives rise to *g*_┴_ = 2.33, *g*_‖_ = 2.00 with *A*_┴_^Co^ = 6.6 mT and *A*_‖_Co = 14.7 mT. Such spectra typically exhibit broadening of the *A*_┴_^Co^ lines (23). This is due to the noncolinearity of the *g* and *A* tensors in the corrin plane (*i.e.*, *x* and *y* directions combined as perpendicular) together with an element of dislocation strain. The slight increase in *A*_‖_^Co^ relative to *Np*RdhA (14.3 mT) represents a trivial change in the orientation of the chloride ion relative to the Co^2+^. Some indication of the chlorine (I = 3/2 for both ^35^Cl and ^37^Cl in an approximately 3:1 abundance ratio, respectively; (24)) superhyperfine coupling quartet observed for *Np*RdhA is also evident, although the aforementioned issues with noncollinearity of *g* and (super)hyperfine tensors render the quartet asymmetric in splitting and intensity.

The addition of 35-DB-4-OH as a substrate results in an EPR spectrum manifesting a very complex hyperfine splitting due to the presence of, and apparent superhyperfine coupling of Co^2+^ to, two bromine atoms, Figure 9*C*. Bromine (^79^Br, ^81^Br), like chlorine, has I = 3/2, but it has a nuclear magnetic moment approximately three times that of chlorine. In addition, it has a nuclear quadrupole moment approximately four times that of chlorine (24). It is these increases that result in the highly complicated splitting pattern and, together with the aforementioned signal to noise and tensor noncollinearity issues, allow only qualitative comparisons between substrate bound and free enzyme. Despite this, it is clear that both *Jt*RdhA^MBP^ and *Np*RdhA, Figure 9*D*, undergo the same spectral changes on binding this substrate. These changes include an increase in *A*_‖_^Co^ to ∼16 mT, an apparent increase in *g*_┴_. Studies with chlorinated substrates binding to *Np*RdhA presented in (25) reveal that this apparent increase in *g*_┴_ actually arises from a weakly rhombic anisotropy, with *g*_x_ = 2.48 and *g*_y_ = 2.22 observed for binding of the chloro- analogue, 35-DC-4-OH. Extensive ^79/81^Br superhyperfine splitting in *g*_‖_ produces many lines with correspondingly low intensity in Figure 9, *C* and *D*. If 35-DB-4-OH simply removed the chloride ion from the cob(II)alamin then the *g*_‖_ lines should sharpen due to the loss of the ^35/37^Cl superhyperfine splitting and appear comparable with the chloride-free *Np*RdhA spectrum shown in Figure 9*B* inset. Spectra A through D are marred by an artefact around *g* = 2 that arises from the subtraction of a [3Fe-4S]^1+^ signal (26) that amounts to less than 4% of the total EPR signal. The isolated [3Fe-4S]^1+^ signal, obtained through difference spectroscopy from samples run at various temperatures, is shown in the inset to Figure 9*A*.

The addition of NADPH to *Jt*RdhA^MBP^ in the presence of the substrate leads to the loss of the cob(II)alamin signal in the EPR spectrum at 30 K, Figure 9*E*. A new signal appears attributable to the [2Fe-2S]^1+^ state of the C-terminal JtPDOR domain 2Fe-2S cluster (27). This domain contains the characteristic CxxxxCxxC-x_n_-C “plant type” ferredoxin motif (28). Additional spectral contributions arise from the semiquinone radical state (FMNH^•^ from the linewidth and “wings” to high and low field, but we cannot be definitive under these conditions) of the PDOR-module FMN cofactor (29), inset to Figure 9E. The [2Fe-2S]^1+^ spectrum is clearly a rhombic spectrum with two discernible *g* values, *g*_1_ = 2.05 and *g*_3_ = 1.87, *g*_2_ being obscured by the flavin semiquinone signal at *g* = 2.00. The apparent lack of significant dipolar broadening evident in either the [2Fe-2S]^1+^ or the flavosemiquinone spectrum is also a feature of PDOR proteins (30). At 30 K no [4Fe-4S]^1+^ spectrum is detectable due to lifetime broadening. The loss of the cob(II)alamin spectrum indicates conversion to either the EPR-silent cob(I)alamin or cob(III)alamin species.

### *In vivo* dehalogenase activity studies

**Figure 10:**
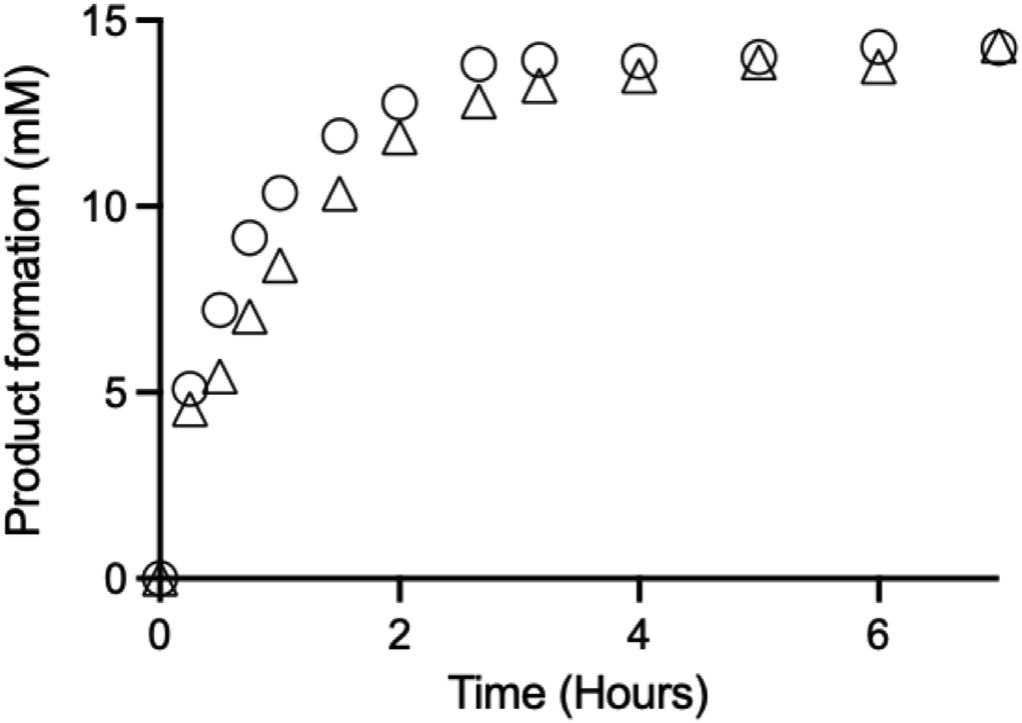
**Whole cell activity of *Jt*RdhA + BtuCEDFB (O) and *Jt*RdhA**^**MBP**^**+ BtuCEDFB (Δ).** Assays contain 20 mM 3,5-dibromo-4-hydroxybenzoic acid and 50 mM glucose in 50 mM Tris-base pH 7.7, 50 mM KCl. Whole cells were standardized to a final *A*_600_ of 15 and *left* shaking at 30 °C. Samples taken at the times shown and product formation (3-B-4OH and 4-OH benzoic acid) determined by HPLC.

*E. coli* HMS174(DE3) cells cotransformed with BtuCEDFB were induced overnight to express *Jt*RdhA^N^ or *Jt*RdhA^MBP^ and used to start whole cell assays. Figure 10 shows the time course for 3B-4-OH product formation as a function of time. Enzyme activity with *Jt*RdhA^N^ or *Jt*RdhA^MBP^-expressing cells was comparable and plateaued after approximately 3 h with about 60% substrate conversion. BtuCEDFB expressed in *E. coli* HMS174(DE3) alone served as a negative control and no activity was detected.

**Figure 11:**
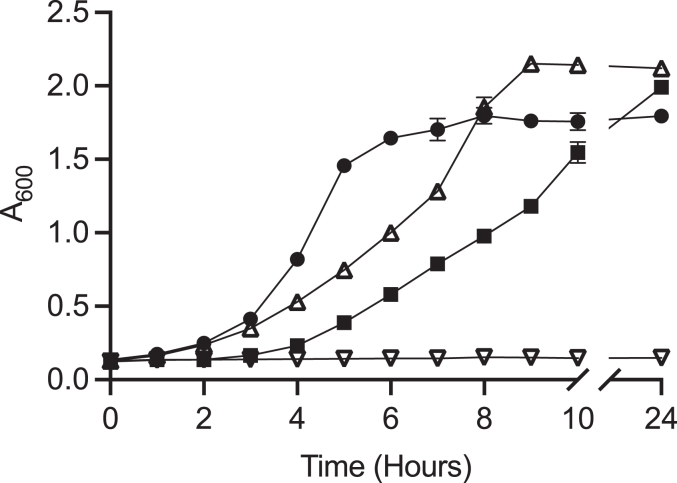
***Jt*RdhA mediated *E. coli* W catabolism of brominated hydroxyphenylacetic acid.** Time course for *E. coli* W-pBbE1k-*Jt*RdhA (∎) and *E. coli* W-pBbE1k-*Jt*RdhA^MBP^ (●) growth on minimal medium with 10 mM 3-bromo-4-hydroxyphenylacetic acid (3Br-4-OHPA) added. *E. coli* W-pBbE1k-RFP, containing the red fluorescent protein (RFP) was used as a negative control (▲) for cell growth and hydroxyphenyl acetic acid was used in place of 3Br-4-OHPA as a positive control (▼). Error bars represent SEM, n = 2.

*E. coli* W was used in order to show whether *Jt*RdhA^MBP^ could convey the ability to *E. coli* to grow on halogenated compounds as, in contrast to K12 strains, this strain possesses all the genes required to grow on 4-hydroxyphenylacetic acid (31). Indeed, *E. coli* W harboring a control plasmid expressing red fluorescent protein was able to grow using 10 mM 4-hydroxyphenylacetic acid as the sole carbon source, whereas no growth was observed with 10 mM 3-bromo-4-hydroxyphenylacetic acid (Fig. 11). However, *E. coli* W harboring a *Jt*RdhA^MBP^ expression plasmid was able to grow on both 4-hydroxyphenylacetic acid and 3-bromo-4-hydroxyphenylacetic acid. After an initial lag phase, *Jt*RdhA^MBP^-expressing cell growth on 3-bromo-4-hydroxyphenylacetic occurred at a rate comparable with the red fluorescent protein–expressing cells with 4-hydroxyphenylacetic acid, reaching a similar cell density.

## Discussion

A functional full-length self-sufficient catabolic reductive dehalogenase has been recombinantly expressed in *E. coli* for the first time. *Jt*RdhA^MBP^ was purified with a large proportion of its many cofactors fully incorporated, with high levels of cobalamin incorporation requiring coexpression of pBAD42-BtuCEDFB. The purified enzyme exhibits rapid substrate turnover when supplied with NADPH, exhibiting *k*_cat_ = 600 ± 8 min^−1^ for the best substrate 35-DB-4-OH. Direct comparison with the kinetics of *Np*RdhA is difficult as the latter is reported for a nonphysiological electron transport system (5). At 1:1:1 FMN:2Fe-2S:cobalamin ratio (mimicking the *Jt*RdhA^MBP^), the turnover number is of the order of 1 min^−1^ (5). Furthermore, *Jt*RdhA^MBP^ exhibits very tight coupling of NADPH oxidation to substrate dehalogenation, with a 1:1 NADPH oxidation:substrate reduction (*i.e.*, essentially “leak free”) observed even under aerobic conditions. This contrasts to the ratio of 3.5:1 observed for *Np*RdhA driven by nonphysiological redox partners.

Despite a relatively modest increase (∼15%) in covalent radius from chlorine to bromine, *Jt*RdhA^MBP^ shows an exclusive preference for brominated phenolic compounds but accepts a wide range including the flame retardant TBBPA. Indeed, the chloro-compounds tested act as competitive inhibitors, with 35-DC-4-OH exhibiting a K_i_ very similar to the K_M_ for the analogous 35-DB-4-OH brominated substrate. This suggests the inability to convert chlorinated analogues is not due to a lack of binding but rather due to large differences in the thermodynamics and/or kinetics of the reaction when compared with organobromines. The product complex includes the halide ion, but the ionic radius of bromine is only ∼8% larger than that of chlorine and so this too seems unlikely to be a driver for specificity/activity. Electronegativity differences are also relatively small with chlorine sitting at 3.0 on the Pauling scale (which runs 0–4) with bromine at ∼2.75. Likewise, reduction potentials (reductive dehalogenation) do not differ greatly (∼50 mV) between dibromo- and dichloro- aromatic compounds at pH 7 (32). The enzyme also shows a marked preference for dibrominated substrates with *k*_cat_/K_M_ about 4-fold greater for 35-DB-4-OH (31.6 μM^−1^ min^−1^ corrected for cobalamin content) than for 3-B-4-OH (7.8 μM^−1^ min^−1^ corrected for cobalamin content). Sequence alignment and the corresponding AlphaFold model confirm most of the active site residues are strictly conserved between *Np*RdhA and *Jt*RdhA, including F291, S422, M423, Y426, K488, and R552 (*Np*RdhA sequence numbering, with only minor conservative changes occurring near the second bromine binding site [Fig. 2]).

**Figure 12:**
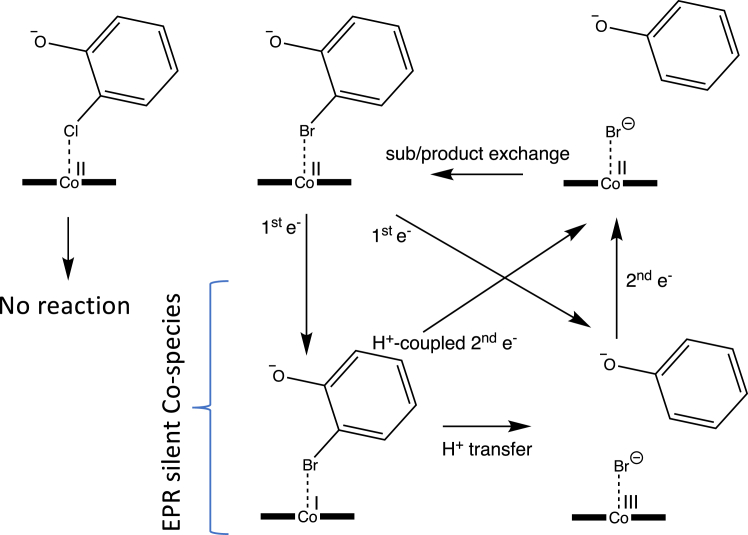
***Jt*RdhA mechanistic insights.** Schematic representation of plausible *Jt*RdhA mechanism under steady-state conditions (excess NADPH). Only for bromo- (or iodo-)phenolic substrates, the Co(II) resting state is replaced by an EPR silent species, suggesting accumulation of either Co(I) and/or Co(III) species, prior to the second electron transfer process that would yield the Co(II) product complex. It remains unclear what the exact nature of the EPR silent Co species is and what the sequence of events following the first electron transfer is. EPR, electron paramagnetic resonance.

In the absence of an X-ray crystal structure of *Jt*RdhA, EPR spectroscopy reveals a cob(II)alamin cofactor in a very similar protein environment to that provided by *Np*RdhA. Like other members of the class III family, the Co^2+^ ion is not coordinated by the DMB ligand or by a ligand (presumed His) from the protein, and like *Np*RdhA, a chloride ion occupies the α-axial position when present in the buffer. This, plus the four equatorial corrin pyrrole nitrogens, allows designation of five-coordinate base-off cobalamin. The chloride ion is displaced by substrate, 3,5-dibromo-4-hydroxybenzoic acid, and the substrate bromine atoms (with I = 3/2) are close enough to the Co^2+^ ion for hyperfine interaction to occur (less than 4 Å). Under turnover conditions, *Jt*RdhA exhibits no cob(II)alamin signal at all, suggesting very effective reduction to the EPR-silent cob(I)alamin or cob(III)alamin species. The only signals observed (at 30 K) are substoichiometric [2Fe-2S]^1+^ and flavin semiquinone (FMNH^•^) spectra. Although quantitation of overlapped signals is difficult, these signals appear to be in a 1:1 ratio and account for 10 to 15% of centers. This suggests a rapid “splitting” of the initially transferred hydride to give a stable [2Fe-2S]^1+^ + FMNH^•^ state rather than [2Fe-2S]^2+^ + FMNH_2_. Therefore, it appears that under turnover conditions the second electron transfer to the corrinoid (which would result in the Co(II)-halogen ion product) is rate limiting, or gated by another process following the initial eT transfer (Fig. 12). Further studies will be required to resolve the exact sequence of events. Unlike *Np*RdhA, *Jt*RdhA appears unable to reduce chlorinated compounds, and it remains to be determined what is the structural and/or mechanistic basis for this. With only one example studied in detail, it remains unclear whether the Rdh-PDOR fusion self-sufficient enzymes substrate scope is limited to brominated compounds.

Dehalorespiring bacteria have long been studied with a view to exploiting their metabolic potential for the bioremediation of environments contaminated with organohalide compounds (33). However, many naturally dehalorespiring organisms are slow growing and require anaerobic conditions that can limit applicability. In addition, many natural dehalorespiring bacteria have high substrate specificity and in the case of polyhalogenated compounds may only catalyze partial dehalogenation, such that their resulting products remain toxic, or in some cases more toxic than the original substrate (34, 35). Similarly, even in cases where complete dehalogenation may be achieved, the remaining organic backbone may still pose environmental issues. An example of the latter is the fire retardant TBBPA, where the dehalogenated product, bisphenol A (BPA) possesses estrogen mimicking properties with obvious implications to both human health and the environment (36). Complete mineralization of TTBPA has been achieved with a mixed culture of naturally derived organisms (37). However, the anaerobic reductive dehalogenation step and the O_2_-dependent BPA degradation step require cycling between anaerobic and aerobic conditions. We have shown the oxygen tolerance of *Jt*RdhA^MBP^ and its use of a universal biologically available reductant (NAD(P)H) allow complete mineralization and growth of an engineered organism on a halo-organic compound (3-bromo-4-hydroxyphenylacetic acid) under aerobic conditions when heterologously expressed in *E. coli* strain W. While 3-bromo-4-hydroxyphenylacetic acid is not a compound of significant environmental concern, the proven ability of *Jt*RdhA to catalyze the successive debromination of TBBPA to BPA suggests that this enzyme could support complete degradation and growth on TBBPA when in the presence of a BPA utilizing pathway.

The *in vivo* studies reported here demonstrate the efficacy of dehalogenation through recombinant expression of functional *Jt*RdhA in nonnative host bacteria. They provide the basis for the biotechnological application of whole cell reactors that employ such bacteria in bioremediation.

## Experimental procedures

### Cloning of *J. thermophila* RdhA

The gene encoding *Jt*RdhA (WP_104007082) was codon optimized to remove codons that were rare in *E. coli* and was synthesized (GenArt). For expression of C-terminally His-tagged *Jt*RdhA in *E. coli* the gene was amplified with CloneAmp (Clontech) and the primers JtpET30F/JtpET30R (Table S1) and the resulting PCR products were cloned into *Nde*I/*Xho*I-linearized pET30a using Infusion HD (Clontech). To produce *Jt*RdhA with an N-terminal His-MBP-tag (*Jt*RdhA^MBP^) the gene was amplified with primers JtpOPINMF/JtpOPINMR (Table S1) (38) and cloned into *Nco*I/*Hind*III-linearized pOPINM, an *E. coli* expression plasmid encoding an N-terminal MBP tag to aid solubility and purification (39).

Once the sequence of the desired insert was confirmed, the corresponding purified plasmid was transformed into *E. coli* HMS174(DE3). Constructs containing RdhA domains were cotransformed with plasmids encoding BtuCEDFB, a cobalamin transporter system required to provide sufficient levels of B12 *in vivo* (15).

For organohalide-dependent growth experiments, *Jt*RdhA was cloned into pBbE1K (38) allowing induction of protein expression using the *lac* promoter. Primers (Table S1) were designed for amplification of both the vector and insert providing 15- to 20-base pair complementary overhangs for DNA assembly using NEBuilder HiFi DNA Assembly kit (NEB). The *Jt*RdhA insert and pbBE1K vector backbone were PCR amplified using Phusion polymerase and 0.5 μM of the respective forward and reverse primers. Successful PCR reactions were cleaned up, using the Macherey Nagel NucleoSpin Gel and PCR Clean-up kit, and vectors assembled using the NEBuilder HiFi DNA Assembly kit (NEB) and transformed into NEB5α. DNA constructs were confirmed by DNA sequencing before transformation into *E. coli W*. Competent *E. coli W* cells were made and transformed as described in (40).

### Large-scale heterologous expression and purification of *Jt*RdhA and *Jt*RdhA^MBP^

*E. coli* transformants were grown overnight in 150 ml Terrific Broth medium containing 0.4% glycerol and phosphate buffer (0.017 M KH_2_PO_4_, 0.072 M K_2_HPO_4_, pH 7.2). To each flask antibiotic was added appropriate to the vector resistance at a standard concentration (50 μg/ml for kanamycin, ampicillin, carbenicillin, and spectinomycin). This culture was used to inoculate 22 L sterile Terrific Broth medium in a Type NLF 22, 30-L BioEngineering fermenter at 37 °C, with agitation at 200 rpm and a compressed air gas flow of 30 L per min until mid-log phase was attained. The medium was then cooled to 20 °C after which 1 μM vitamin B12 and 50 μM ammonium iron (II) sulfate were added, and protein expression was induced with 0.2 mM IPTG. The cell culture was left for 16 h with shaking at 200 rpm, after which time cells were harvested *via* centrifugation at 6000 rpm for 10 min at 4 °C in a Beckman Coulter, Avanti J26-XP centrifuge fitted with a JLA 8.1000 rotor. Cells were frozen and stored at −80 °C until further use.

Harvested cells were thawed under a nitrogen atmosphere and resuspended in anoxic 50 mM Tris-base pH 8, 200 mM NaCl, 10% glycerol at a ratio of 1:3 (weight/volume). Once cells were defrosted, RNase, DNase, and SigmaFast EDTA-free protease inhibitor cocktail were mixed into the homogenous cell solution. Cells were lysed by passage through a French pressure cell under 1250 psi pressure with sample and collection bottles constantly purged with nitrogen. Lysed cells were collected on ice. Cell lysates were decanted anaerobically into 70 ml polycarbonate ultracentrifuge tubes and clarified by ultracentrifugation in a Beckman Coulter, Optima L-100 XP Ultracentrifuge at 185,000*g* for 1 h at 4 °C.

The supernatant was applied to a Ni-NTA agarose drip column (Qiagen) in the anaerobic chamber. The column was washed successively with 5 column volumes of 50 mM Tris-base pH 8, 10% glycerol, 200 mM NaCl supplemented with first 10 mM and then 40 mM imidazole. The protein was eluted with 250 mM imidazole in the loading buffer. Imidazole was removed using a PG6D desalting resin (Bio-Rad). Samples from the lysate, wash, and elution fractions were analyzed by 4 to 20% SDS-PAGE to determine fractions containing the protein of interest.

Partially pure *Jt*RdhA^MBP^ protein was applied to amylose affinity resin and eluted with 50 mM Tris-base pH 8, 10% glycerol, 200 mM NaCl, and 10 mM maltose. When necessary, the protein was subjected to a final aerobic purification step on a Superdex S-200 (0.4 ml min^−1^, 120 ml S200 16/600Gl, GE Healthcare) gel filtration column equilibrated in 50 mM Tris-base pH 8, 150 mM NaCl, 10% glycerol on an AKTA Purifier. The heterologous expression and purification of spinach ferredoxin and *E. coli* flavodoxin reductase was performed exactly as described in (11).

### UV-Visible and EPR spectroscopy

UV-visible spectra were measured between 200 and 800 nm in a Cary 50Bio UV-visible spectrophotometer. All samples were baseline corrected using appropriate buffer. Purified protein concentration was determined by its absorbance at 280 nm using its extinction coefficient (determined by primary amino acid sequence using the ExPASy ProtParam tool) or by the Lowry method described in (16).

*Jt*RdhA protein samples for EPR were prepared either as isolated or reduced with 25 μM 5-deazariboflavin and 2 mM EDTA under a blue LED for 1 h before being transferred into 4-mm Suprasil quartz tubes under anoxic conditions, sealed with a No. 9 Suba-seal rubber stopper, and frozen in liquid nitrogen. X-band (9–9.5 GHz; IEEE definition 8–12 GHz) continuous-wave EPR spectra were obtained using Bruker ELEXSYS E500/E580 spectrometers at the UK EPSRC National Service for Electron Paramagnetic Resonance Spectroscopy and the Manchester Institute of Biotechnology. Samples frozen (liquid nitrogen, 77 K) in 4-mm o.d. tubes were placed in a Bruker Super High Q (ER4122SHQ) cavity resonator and cooled to spectrum acquisition temperature using liquid helium flow through an Oxford Instruments ESR 900 cryostat. Signal quantitation was *via* double integration and comparison with Cu^2+^-EDTA standards. Experimental parameters and temperatures are as stated in the figure captions.

### *Jt*RdhA activity assays

Reaction mixtures (500 μl) including 10 mM NADPH, 1 μM *Jt*RdhA, 50 mM Hepes pH 7.7, 100 mM NaCl, and 5 mM organohalide substrate were set up in an anaerobic glovebox using crimp seal vials. Vials were removed from the glovebox and incubated at 30 °C and 750 rpm. After incubation, assays were terminated by the addition of 50 μl of 30% tricarboxylic acid cycle before spin centrifugation in a benchtop microfuge at 13,200 rpm and 4 °C to sediment the precipitated reaction components. The supernatant was taken for HPLC analysis using an Agilent 1200 Infinity Series HPLC instrument equipped with a UV detector. The stationary phase was a Kinetex 5 μm C18 100 Å column, 250 × 4.6 mm. The mobile phase was acetonitrile/water (50:50) with 0.1% TFA at a flow rate of 1 ml min^−1^ for 10 min, and detection was performed at the wavelength relevant for the substrate under investigation. Standard curves for each substrate and product (0.1–2 mM), when available, were used to compare product peaks obtained in each assay and to calculate turnover rates.

NADPH consumption was measured by following the decrease in absorbance at 340 nm (ε = 6.22 mM^−1^ cm^−1^) using a Cary 50Bio UV-visible spectrophotometer over 5 min and fitted to a linear rate. Assays were performed anaerobically and contained 1 to 500 μM substrate, 200 μM NADPH, 50 mM KCl, and 0.5 or 1 μM dehalogenase in 50 mM Tris-base.

The inhibition of 3,5-dibromo-4-hydroxybenzoic acid (35-DB-4-OH) reduction was monitored by following NADPH consumption at 340 nm at nonsaturating substrate concentrations (0.1, 0.03, and 0.015 mM) while varying the concentration of 3,5-dichloro-4-hydroxybenzoic acid (35-DC-4-OH) over the range 0.01 to 0.15 mM together with a control lacking inhibitor. Assays were performed anaerobically and contained 200 μM NADPH and 0.5 μM *Jt*RdhA in 50 mM Tris-base pH 7.7, 50 mM KCl.

The noncognate reductase system assays were as reported (11) with 1 μM *Np*RdhA, 400 μM spinach ferredoxin, and 40 μM *E coli* flavodoxin reductase.

TBBPA dehalogenation was performed under an N_2_ environment with reactions left shaking at 30 °C. TBBPA (2 mM) was dissolved in anaerobic iso-octane, and assays were performed as biphasic reactions in sealed vials. The organic layer was removed and analyzed by HPLC with a Zorbax SB-C18 column and isocratic elution with acetonitrile–water (70:30 v/v).

Electrospray ionization (negative mode) was performed, and the products were identified by the mass spectrum at the corresponding retention times; TBBPA *m/z* = 543.75, tribromo *m/z* = 464.84, dibromo *m/z* = 385.93, and the mono-brominated reaction intermediate *m/z* = 306.02.

For whole cell assays, constructs containing *Jt*RdhA were cotransformed with plasmids encoding BtuCEDFB into *E. coli* HMS174(DE3) cells and grown on Terrific Broth at 37 °C to an *A*_600_ of 0.8. Cells were then induced with 0.2 mM IPTG and grown overnight at 20 °C in the presence of 1 μM vitamin B12, 1 μM hydroxocobalamin, and 50 μM ammonium iron (II) sulfate. After induction cells were resuspended in a small volume of 50 mM Tris-base pH 7.7, 50 mM KCl sufficient to give a final *A*_600_ of 25. Assays were conducted at 30 °C and contained 50 mM glucose, 25 mM 35-DB-4-OH, and a final whole cell *A*_600_ of 15. Samples were taken for HPLC analysis at various time points to determine product levels.

For *E. coli* W whole cell growth on organohalides, cells were grown during the day in 5 ml minimal medium supplemented with 10 mM glucose. Overnight cultures of 50 ml minimal medium containing 10 mM glucose or 10 mM hydroxyphenyl acetic acid were inoculated with 0.1 ml day culture and left to grow overnight at 37 °C. On the next day, cultures were spun and washed twice with minimal medium before finally being resuspended in 5 ml minimal medium. Sufficient culture was added to 50 ml minimal medium containing 10 mM halogenated substrate, 50 μM ammonium iron (II) sulphate, 1 μM vitamin B12, and 50 μM IPTG to give a starting *A*_600_ = 0.1. Growth was monitored at regular intervals while shaking at 30 °C and 200 rpm.

### Iron, B12, and FMN cofactor content quantification

Iron content was determined colorimetrically with bathophenanthroline after acid denaturation exactly as reported by Collins *et al.* (11).

B12 concentration was estimated either by cyanide extraction or spin quantification of the Co(II) signal *via* EPR. Cyanide extraction involved mixing equal volumes of protein of known concentration, typically 100 to 150 μM, and potassium cyanide (10 mM) and heat denaturation for 20 min at 80 °C to release the cobalamin. Measurement of the cyanocobalamin UV-visible spectra and quantification of its concentration *via* its 550 nm peak (ε = 8.7 mM^−1^ cm^−1^) allowed determination of the protein:cobalamin ratio.

Flavin content was determined with an Agilent 1200 Infinity HPLC with a UV detector attached. The stationary phase used was a Kinetex 5μ C18 100.70 column, 25 × 4.6 mm. The mobile phase was 80% water and 20% acetonitrile containing 0.1% trifluoroacetic acid at flow rate of 1 ml min^−1^ for 10 min. Protein samples of known concentration were heat denatured at 80 °C for 15 min followed by removal of precipitate by centrifugation. Flavin concentrations were determined from the integration of peaks observed at 450 nm with comparison with a standard curve.

### *Jt*RdhA structure modeling

The *Jt*RdhA model was predicted using AlphaFold (41) *via* the ColabFold (v1.5.2) notebook (42). The highest ranked model was taken as a starting point for cofactors to be added by aligning each individual domain of the *Jt*RdhA model against the most homologous crystal structure. For the dehalogenase domain, the *Jt*RdhA model was aligned with the *Np*RdhA structure (Protein Data Bank: 6ZY1, rmsd = 0.9 Å), allowing placement of the corresponding cobalamin cofactor and [4Fe-4S] clusters. For the oxidoreductase domain, the *Jt*RdhA model was aligned with a self-sufficient cytochrome P450 structure (Protein Data Bank: 6KBH, rmsd = 2.2 Å) to generate the corresponding FMN and [2Fe-2S]. Amino acids interacting with these cofactors were checked for proper orientation, and the rotamer tool of Chimera 1.16 (43) was used to improve cofactor/protein interactions.

## Data availability

Data are provided either within the article or as part of the supporting information.

## Supporting information

This article contains supporting information.

## Conflict of interest

The authors declare that they have no conflict of interest with the contents of this article.
