# Supplementary material for: Efficient NADPH-dependent dehalogenation afforded by a self-sufficient reductive dehalogenase
Source: J Biol Chem. 2023 Jul 25;299(9):105086. doi: 10.1016/j.jbc.2023.105086 (PMC10463259; doi:10.1016/j.jbc.2023.105086)
Supplement: Supporting Figures S1–S4 and Table S1 [file mmc1.docx]

**NADPH-dependent reductive dehalogenation afforded by a self-sufficient reductive dehalogenase**

Karl Fisher^1$^, Tom Halliwell^1$^, Karl A.P. Payne^1^, Gabriel Ragala^1^, Sam Hay^1^, Stephen E.J. Rigby^1^, David Leys^1^*

^1^Manchester Institute of Biotechnology, University of Manchester, 131 Princess Street, Manchester, M1 7DN, UK

Running title: *Self-sufficient* RdhA

*To whom correspondence should be addressed: Prof. David Leys, Manchester Institute of Biotechnology, University of Manchester, Princess Street 131 Manchester, M1 7DN, UK. Tel: 0044 161 306 51 50; Email: david.leys@manchester.ac.uk

**^$^**These authors contributed equally to this work

**Supplementary material**

**Table S1, Figures S1-S4**

**
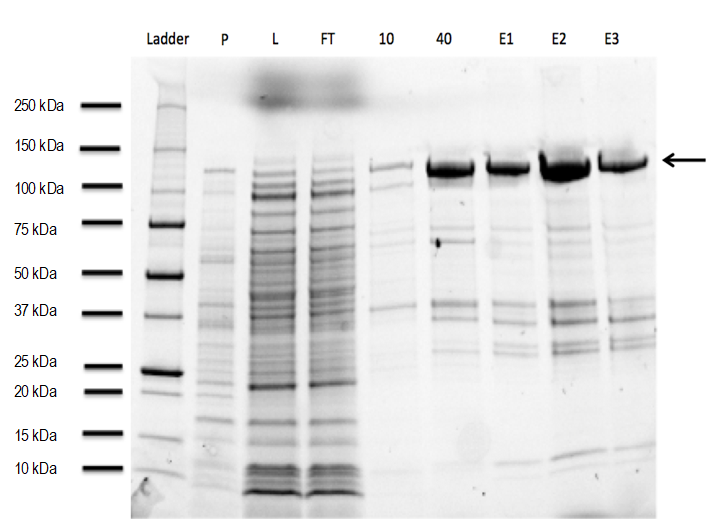
**

**Figure S1 Anaerobic Gravity Flow Ni-NTA Purification of JtRdhA^N^.** Anaerobic Gravity Flow Ni-NTA Purification of Jt^N^- Protein purified in anaerobic chamber at 20°C. P = pellet, L = load, FT = flow through of cleared lysate, 10 = 10 mM imidazole wash fraction, 40 = 4 0 mM imidazole wash fraction and E1-E3 = 200 mM imidazole elution fractions.


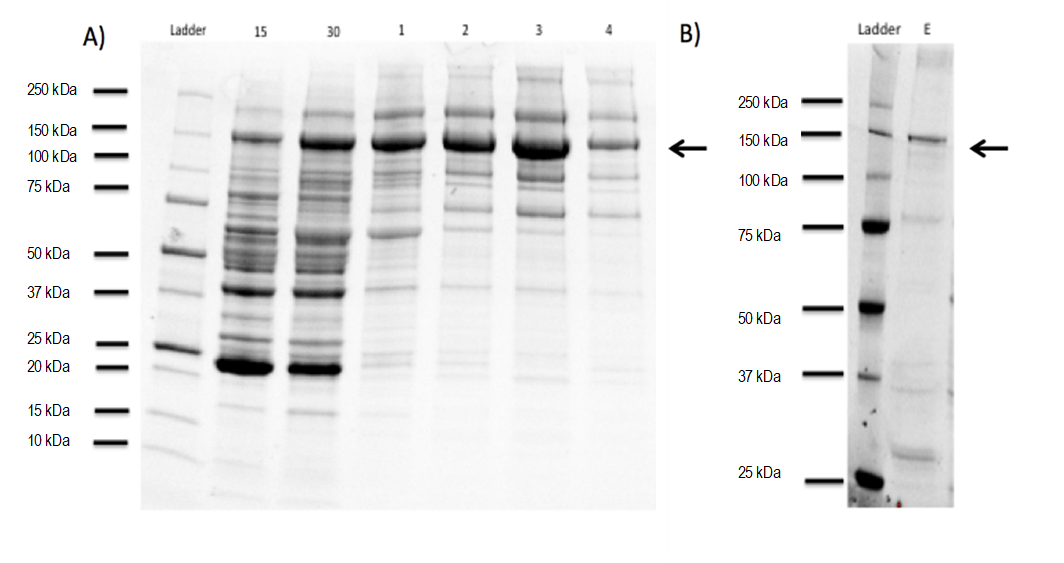


**Figure S2: SDS-PAGE of anaerobic *Jt*RdhA^MBP^ purification.** A) Gravity flow Ni-NTA: 15 mM imidazole wash, 30 mM imidazole wash, 1-4 = 10 mL elution fractions with 250 mM imidazole. B) Size exclusion chromatography purification of elution fraction from A. E = sample from elution peak.

**
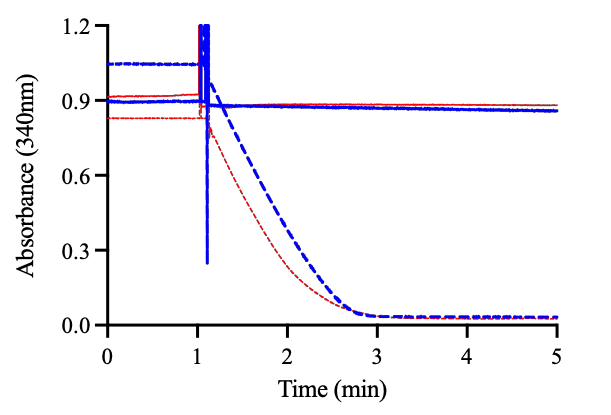
**

**Figure S3: Comparison of *Jt*RdhA^MBP^ NADPH consumption under aerobic and anaerobic conditions.** *Jt*RdhA^MBP^ dehalogenase activity was measured with 35-DC-4OH (solid line) acid and 3,5-DB-4-OH (dashed line) by monitoring NADPH consumption at 340 nm under air (blue line) or nitrogen (red line) at 20° C. All reactions were performed in triplicate and contained 0.5 μM *Jt*RdhA^MBP^, 0.2 mM NADPH and 0.25 mM substrate. Assays were initiated after ca 1min by the addition of *Jt*RdhA^MBP^.

**
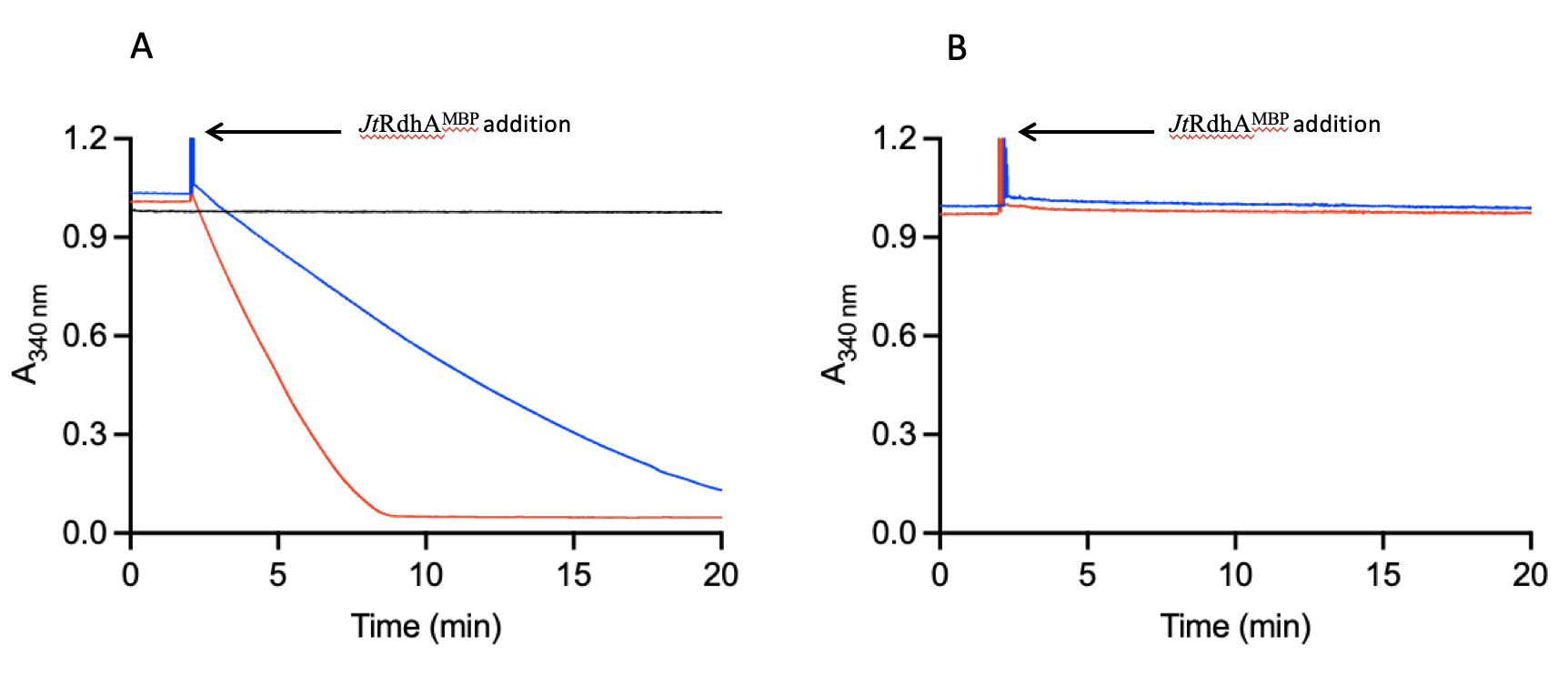
**

**Figure S4: Comparison of *Jt*RdhA^MBP^ NADPH consumption with 3,5 DC-4-OH under aerobic and anaerobic conditions.** *Jt*RdhA^MBP^ dehalogenase activity was measured with 3,5 DC-4-OH by monitoring NADPH consumption at 340 nm under air (panel A) or nitrogen (panel B) at 20° C. Panel A shows NADPH usage with no *Jt*RdhA^MBP^ added (black line), *Jt*RdhA^MBP^ (blue line) and *Jt*RdhA^MBP^ with 3,5, dichloro-4-hydroxybenzoic acid (red line). Panel B shows *Jt*RdhA^MBP^ with and without 3,5,DC-4-OH (blue and red lines respectively) under anaerobic conditions.

Where stated reactions contained 5 μM *Jt*RdhA^MBP^, 0.2 mM NADPH and 1 mM substrate and assays were initiated after ca 1min by the addition of *Jt*RdhA^MBP^
